# Effect of wet clothing removal on skin temperature in subjects exposed to cold and wrapped in a vapor barrier: a human, randomized, crossover field study

**DOI:** 10.1186/s12873-024-00937-8

**Published:** 2024-01-25

**Authors:** Linn Therese Hagen, Guttorm Brattebø, Jörg Assmus Dipl-Math, Øystein Wiggen, Øyvind Østerås, Sigurd Mydske, Øyvind Thomassen

**Affiliations:** 1 Department of Anaesthesia and Intensive Care, Haukeland University Hospital, P.O. Box 1400, Bergen, 5021 Norway; 2 Faculty of health sciences, University of Stavanger, Stavanger, Norway; 3 Mountain Medicine Research Group, The Norwegian Air Ambulance Foundation, Bergen, Norway; 4 Department of Clinical Medicine, University of Bergen, Bergen, Norway; 5 Centre for Clinical Research, Haukeland University Hospital, Bergen, Norway; 6 SINTEF Technology and Society, Preventive Health Research, Trondheim, Norway

**Keywords:** Accidental hypothermia, Vapor barrier, Mountain medicine, Emergency medicine, Wet clothing, Field study, Human, Crossover, Clinical trial, Active external rewarming

## Abstract

**Background:**

Prehospital care for cold-stressed and hypothermic patients focuses on effective insulation and rewarming. When encountering patients wearing wet clothing, rescuers can either remove the wet clothing before isolating the patient or isolate the patient using a vapor barrier. Wet clothing removal increases skin exposure but avoids the need to heat the wet clothing during rewarming. Leaving wet clothing on will avoid skin exposure but is likely to increase heat loss during rewarming. This study aimed to evaluate the effect of wet clothing removal compared to containing the moisture using a vapor barrier on skin temperature in a prehospital setting.

**Methods:**

This randomized crossover experimental field study was conducted in a snow cave in Hemsedal, Norway. After an initial cooling phase of 30 min while wearing wet clothes, the participants were subjected to one of two rewarming scenarios: (1) wet clothing removal and wrapping in a vapor barrier, insulating blankets, and windproof outer shell (dry group) or (2) wrapping in a vapor barrier, insulating blankets, and windproof outer shell (wet group). The mean skin temperature was the primary outcome whereas subjective scores for both thermal comfort and degree of shivering were secondary outcomes. Primary outcome data were analyzed using the analysis of covariance (ANCOVA).

**Results:**

After an initial decrease in temperature during the exposure phase, the dry group had a higher mean skin temperature compared to the wet group after only 2 min. The skin-rewarming rate was highest in the initial rewarming stages for both groups, but increased in the dry group as compared to the wet group in the first 10 min. Return to baseline temperature occurred significantly faster in the dry group (mean 12.5 min [dry] vs. 28.1 min [wet]). No intergroup differences in the subjective thermal comfort or shivering were observed.

**Conclusion:**

Removal of wet clothing in combination with a vapor barrier increases skin rewarming rate compared to encasing the wet clothing in a vapor barrier, in mild cold and environments without wind.

**Trial registration:**

ClinicalTrials.gov ID NCT05996757, retrospectively registered 18/08/2023.

**Supplementary Information:**

The online version contains supplementary material available at 10.1186/s12873-024-00937-8.

## Background

Shivering patients with a core temperature (T_core_) of > 35 °C is cold-stressed, but not hypothermic [1]. Trauma patients with decreased T_core_ have been found to have a higher mortality risk than normothermic trauma patients [2–4]. Cold exposure is extremely uncomfortable and can exacerbate fear, pain, and an overall sense of dissatisfaction [5]. Prehospital care of cold-stressed or hypothermic patients aims to rewarm to- or maintain normothermia to decrease morbidity and mortality, as well as to reduce both the pain associated with freezing and anxiety [6–8]. Feasible low-reading thermometers are often unavailable in the prehospital services [9]. Due to the lack of adequate diagnostic tools, an accurate temperature measurement is often not obtained, and this may lead to an underestimation of the incidence of prehospital accidental hypothermia [2, 10, 11]. Taking this into account, some guidelines recommend that all cold patients should be protected from the environment, and depending on the severity of hypothermia, may also benefit from active external warming [1].

There are four main mechanisms of heat loss: (1) conduction, wherein heat loss occurs from the skin to a solid material that is in contact with the body; (2) Convection, wherein heat loss occurs due to the wind or liquid; (3) Radiation, which is the transfer of heat in the form of electromagnetic energy between two objects that are visible to each other; and (4) Evaporation, wherein heat loss occurs due to wet skin and/or wet clothing [1].

There is a need to clarify the possible thermal disadvantages of packaging a patient in a vapor barrier with wet clothes. Removal of wet clothing will briefly entail exposure of wet skin to the environment before the insulation is applied. However, there will be less moisture inside the wrapping model, requiring thermal energy for water to be converted to vapor. Conversely, wrapping the patient with wet clothing in a vapor barrier avoids skin exposure but leads to more water retention. Although the vapor barrier limits evaporation, the patient expends more thermal energy to heat the water to body temperature. A previous study has showed that the use of either wet clothing removal or the use of a vapor barrier is beneficial in these patients compared to simply wrapping the wet clothing in a woolen blanket, but these studies does not show between wet clothing removal and leaving the clothing on inside a vapor barrier [12].

This study aimed to evaluate the effect on the skin temperature with removal of wet clothing compared with moisture containment using a vapor barrier in a standardized field setting, as well as the effect on thermal comfort and degree of shivering.

## Methods

### Study design and outcome measures

The study was conducted as a crossover clinical trial performed under field conditions. The outcome measures were skin temperature (T_skin_), subjective evaluation of comfort, thermal sensation, and degree of shivering. Alterations in skin temperature are the first compensatory mechanisms for thermal homeostasis and their sensitivity has previously been demonstrated in other studies that evaluated cold-protective measures in prehospital care which used cold air as a cooling mechanism to cool the participants to even lower skin temperatures than those in this study [12, 13]. The T_core_ remained normal throughout the cooling and rewarming phases in these studies; therefore, the T_core_ was not measured in our study.

### Participants and study setting

Participants were recruited from among volunteer healthcare workers and medical students. The inclusion criteria were as follows: age > 18 years, good overall health (American Society of Anesthesiologists Class 1), and no nicotine use. All participants were instructed to abstain from physical exercise and alcohol consumption for at least 24 h before the study. This study was conducted in March 2017 in a specially designed snow cave (22.5 m^3^; 4.9 m × 2.7 m × 1.7 m) that was situated 660 m above the sea level in Hemsedal, Norway. The ambient air temperature and humidity in the snow cave, resting area, and immediately outside the cave were recorded throughout the study day. Written informed consent was obtained from all research participants, and the research project was approved by the Regional Ethics Committee for Medical and Health Research (2017/150/REK Nord).

### Preparation

Participants were randomly assigned to two groups that alternated between resting and testing in a snow cave. The participants arrived in the preparation room before the test, wore only underwear, and had a personal assistant for the entire study. The assistants helped fit the participants with skin thermistors and ensured that they were uniformly dressed before the baseline recordings were obtained. The participants were dressed in a T-shirt, long-sleeved shirt, and trousers (all of which were made of cotton). The wet clothes were prepared by being left overnight in a sealed plastic bag containing 700 mL water on a warm bathroom floor and cooled to room temperature before the experiment. This ensured a uniform distribution of moisture in the clothing. Dry fleece hats and mittens were used to insulate the head and hands, and participants wore identical dry cotton socks and sneakers. All participants walked 100 m from the preparation room to the snow-cave laboratory. Participants were placed in the supine position on a 14-mm sleeping mat (Mammut Bamse Extreme, Mammut Sports Group, Seon, Switzerland) that was laid directly on the snow.

### Instrumentation and measurements

The T_skin_ was measured using thermistors (YSI-400 Yellow Springs Instrument, USA; accuracy ± 0.15 °C) connected to a data logger (Smart Reader Plus 8 ACR Systems Inc., USA). The skin thermistors were placed at seven predefined locations (head, arms, hands, feet, legs, thighs, and trunk). The mean skin temperature (T_mean skin_) was calculated using the Hardy–Dubois formula [14, 15]. Local and overall thermal sensation, thermal comfort, and degree of shivering were measured using a modified Bedside Shivering Assessment Scale (BSAS) which can be found in the Supplemental files 1 and 2 [16–18].

### Interruption criteria

The participants could withdraw from the study at any point without explanation. Moreover, the test was to be terminated if one or more of the skin thermistors recorded a temperature ≤ 10 °C for more than 20 min, based on threshold values from ISO-standards for designing immersion suits [19].

### Experimental procedure

After a 30-minute initial cooling phase in a supine position in the snow cave, the participants either had their wet clothing removed (dry group) or retained their wet clothing (wet group) before being placed in a protective and insulating wrapping model. In the dry group, the participants remained in the supine position while the assistants cut away their clothing in a standardized manner. The cutting procedure was pre-rehearsed and timed such that it was identical for each participant. Starting at the sternal notch, cutting was performed medially from the torso to the waistband. Subsequently, both the sleeves were cut from the wrist to the shoulders and neck. The trousers were cut medially from the waistband down toward both lower extremities up to the ankles. Using a log-roll technique, the assistants rapidly removed the wet clothing from underneath the participants. Both groups were wrapped in a vapor barrier (plastic bubble wrap, TAP Telion-Air-Pac GmbH, Braunschweig, Germany), dry insulation layer (woven cotton blankets, 310 g/m^2^), and windproof thermal rescue bag (Fjellduken Thermo Extreme, Jerven AS, Odda, Norway). The exposure time in the dry group (time from the start of cutting until complete wrapping) was less than 2.5 min (120–138 s) for all participants.

Between each scenario, the participants recovered for a minimum of 120 min in a resting room (22.3 ± 1.2 °C) to avoid crossover effects. Participants were encouraged to eat, drink, rest, and become thermally comfortable during this period. Each participant served as their own control and another scenario was conducted after the resting period. A detailed description of each scenario is provided in Table 1.

**Table 1:** Detailed description of the timing for each scenario

Time elapsed	Time for task	Action	
-30 min	30 min	Resting period	
0 min	Start	Participants meet in the preparation room wearing only underwear	
5 min	5 min	Monitoring equipment placed by the assistants	
7 min	2 min	Assistants help participants dress in prepared wet clothing	
10 min	3 min	Assistants and participants walk 100 m to the snow cave, and participants lie down simultaneously	
40 min	30 min	Cooling period T_skin_ recorded every 60 s and cold discomfort recorded every 9 min by the assistants	
45 min	5 min	(1) Assistants cut clothing simultaneously and wrap participants in the insulating layers using the standard technique	(2) Assistants wrap participants in the insulating layers using the standard technique, leaving the wet clothing on subjects
1 h 15 min	30 min	Passive rewarming: T_skin_ recorded every 60 s and subjective degree of cold discomfort and shivering questioned every 9 min by the assistants	
3 h 15 min	120 min	Participants return to the preparation room, where assistants help them remove the monitoring equipment and dress in dry clothes, followed by rest and recovery	

### Statistical analysis

By using the skin temperature changes observed in a study by Henriksson et al. from 2015 as the reference, power calculations were undertaken and indicated that a minimum of six participants would be needed in a crossover design to identify a temperature difference of 1.2 °C with a standard deviation of 0.8, a power of 0.8, and a significance level of 0.05 when using a paired *t*-test [12, 20]. Considering dropouts (e.g., due to technical issues), eight participants were included in the study. Block randomization with a block size of four was used (i.e., each test run was randomized separately).

Descriptive methods were used to characterize the samples. The interventions were compared with regard to the mean T_skin_ by using ANCOVA with random intercepts per participant for each time point from the baseline. We assumed that there was no crossover effect because we only used physical measures in the main analyses, with a sufficient washout period between runs. The elapsed time until the return to baseline temperature was estimated, and a paired t-test was used to compare differences between the groups. Subjective ratings of thermal comfort, thermal sensation, and the degree of shivering were assessed using graphical and descriptive methods and reported as the mean and 95% confidence interval (CI). All calculations were performed using R 3.4.0 and the package Linear and Nonlinear Mixed Effects Models (nlme) [21, 22]. Illustrations were developed in Matlab 9.0 (Natick, MA, USA).

## Results

Eight volunteers (5 men, 3 women) participated in the study, with a median age of 28.5 (range 21–47) years, median height of 180 (range 168–188) cm, and mean body mass index of 23.0 (range 17.7–33.2) kg/m^2^. The study protocol was followed by all participants, and no measurement errors occurred. No participant withdrew from the study. The temperature in the snow cave was 3.1 °C ± 1.2 °C throughout the day (Figure Supplemental 3).

### Skin temperature

**Fig. 1:**
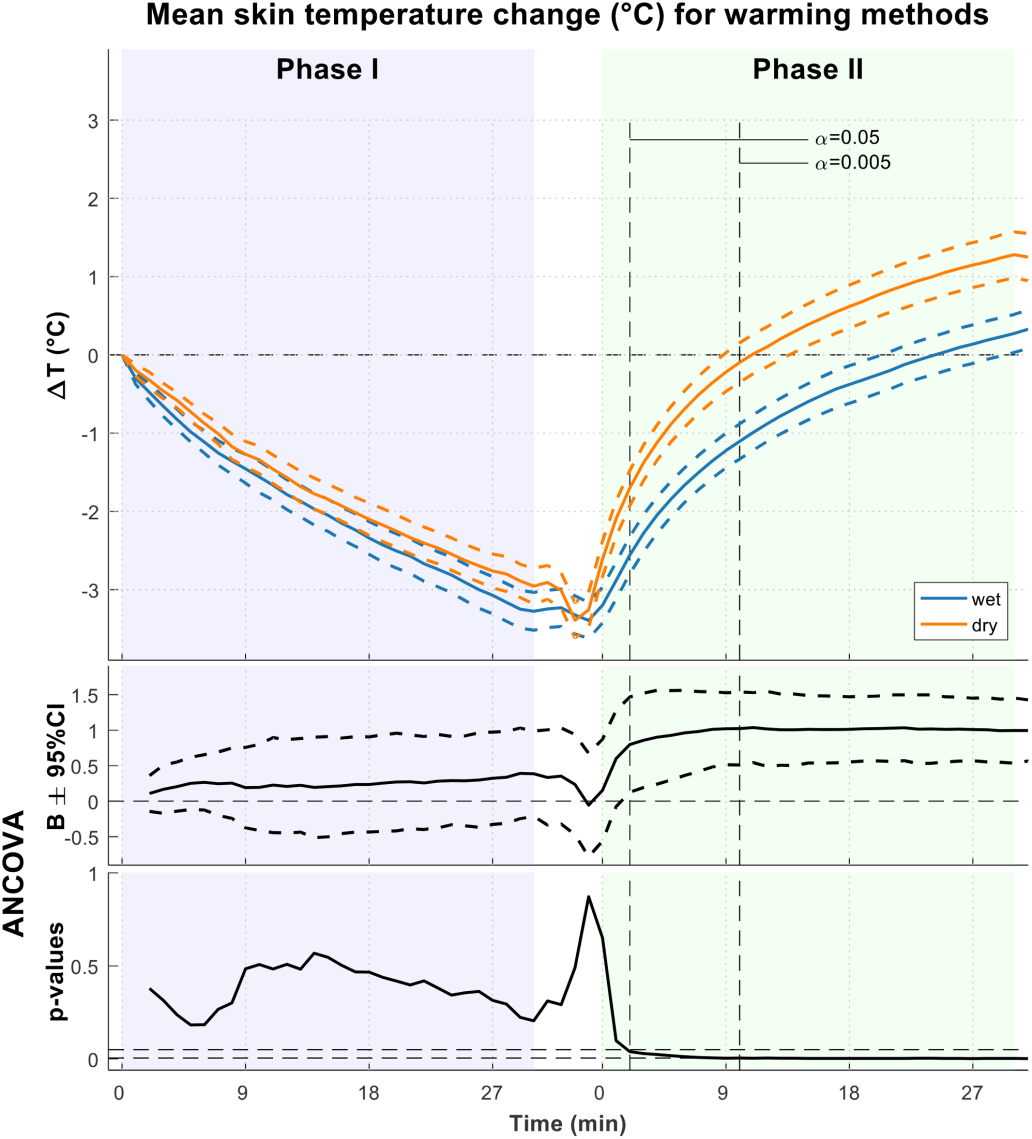
Change in mean T_skin_ for the wet (blue) and dry (red) groups. Coefficient B shows the mean temperature difference between the groups adjusted for baseline

The measured changes in the mean T_skin_ for the two groups are presented in Fig. 1. As expected, both groups experienced a similar decrease in the mean T_skin_ of approximately 3.5 °C during the 30-minute cooling phase (phase 1). The difference observed after 30 min was caused by slight differences at baseline and was not significant (see p-values in ANCOVA). In the dry group, a decrease in the mean T_skin_ was observed during exposure when wet clothes were removed and before wrapping the participant in the insulation. However, a faster increase in the mean skin temperature was observed in the dry group than in the wet group; moreover, after 2 and 10 min, significantly higher values were recorded in the dry group. After 10 min, the mean T_skin_ difference stabilized at 1.0 °C (SD 0.01 °C). The return to baseline temperature occurred significantly faster in the dry group than in the wet group (mean time 12.5 [95% CI 8.3–16.6] minutes versus 28.1 [95% CI 18.8–37.4] minutes).

### Thermal comfort and degree of shivering

**Fig. 2:**
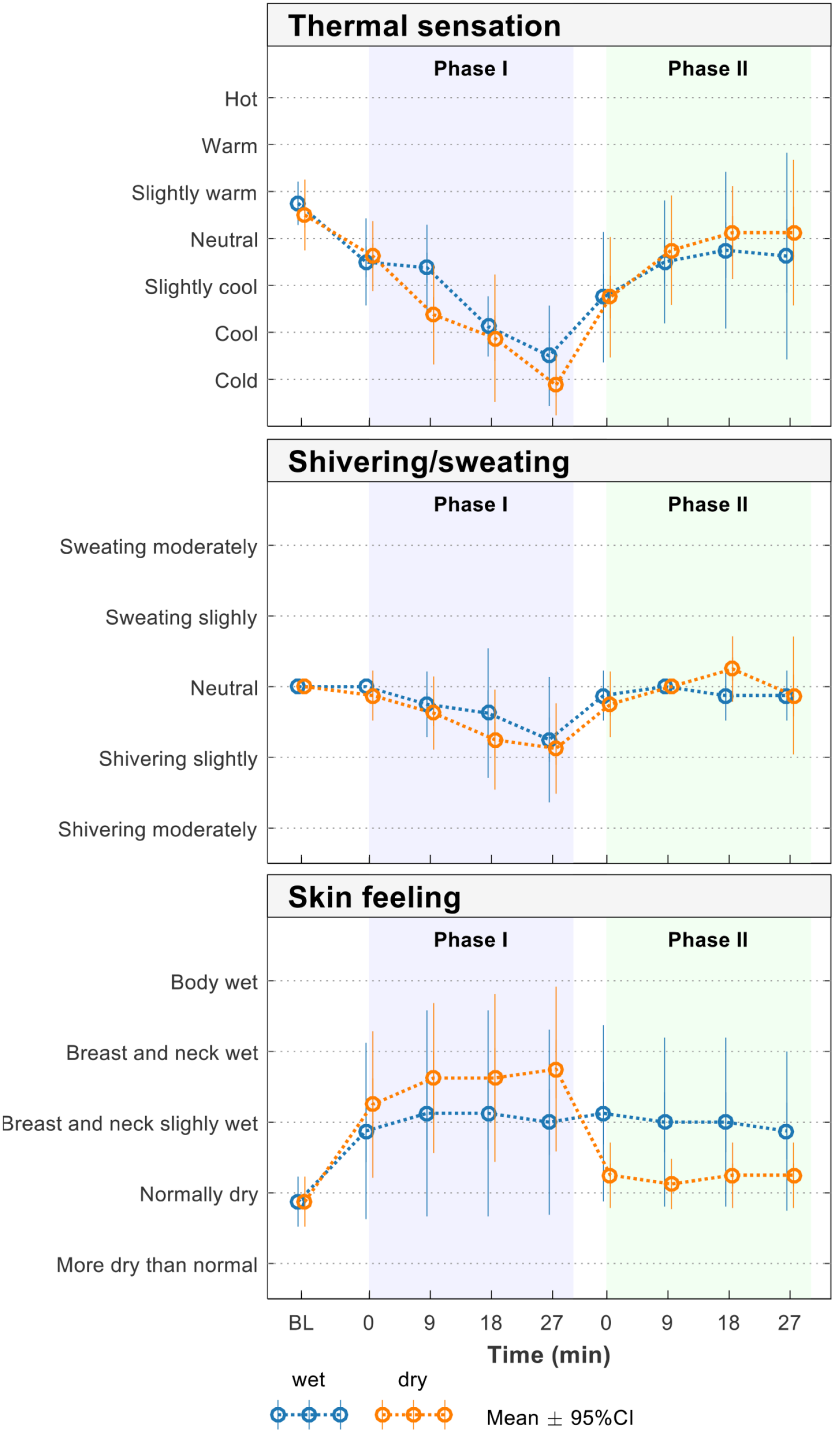
Questionnaire data on thermal sensation, degree of shivering and sweating, and skin feeling among the study participants

We found no significant intergroup differences in terms of subjective thermal comfort or degree of shivering. However, the dry group expressed a feeling of somewhat drier skin immediately after the wet clothing was removed (Fig. 2) whereas the wet group reported no such changes, and their answers remained unchanged from the start to the end of the observation period.

## Discussion

The main finding in this study was that the dry group (removal of wet clothing) had a greater and faster increase in the mean skin temperature (T_skin_) as compared to the wet group (without removal of wet clothing). There was a significant intergroup difference in the T_skin_ at 2 min after the participants were wrapped, and this effect persisted throughout the experiment. Consequently, the dry group experienced a return to baseline temperature in less than half the time required by the wet group. The intergroup difference increased during the first 10 min before stabilizing, thereafter remained similar for the remainder of the experiment.

The largest intergroup difference in the rate of thermal increase occurred in the earliest stages of rewarming; this trend was also observed by Henriksson et al., although the difference was not statistically significant [12]. Our study showed the positive effect of removing wet clothing before applying the vapor barrier and insulation, even when a vapor barrier was available. For all participants, the exposure time after the cooling phase was less than 2.5 minutes, and the T_skin_ rewarming rate was significantly higher in the dry group. Some studies and reviews have warned against the removal of wet clothing as this may result in increased cooling owing to increased exposure [20, 23]. Several environmental factors, such as wind, ambient temperature, and rain/snow may increase the cooling rates. Of note, more severe weather conditions will most likely lead to a more prominent “dip” in T_skin_ during the exposure period than what was demonstrated in our study.

In contrast to the results of this study, Henriksson et al. found no difference between using a vapor barrier and removing wet clothing over a 30-minute period [12]. We assume that the different results of the experiments could be attributed to a slight difference in study design, as the studies had a different room temperature and Henriksson et al. used external heat packs. In our study, a vapor barrier was used in both the dry group and the wet group. Our design enabled us to analyze the removal of wet clothing as a single variable whereas Henriksson was able to compare different methods. We speculate that the faster increase in temperature in the dry group as compared to the wet group during the first few minutes of our experiment was attributable to the increased amount of thermal energy required to heat the additional water that was contained in the clothing of the wet group. Furthermore, we believe that the vapor barrier in the dry group may have been beneficial, as this would prevent evaporation of residual water after the removal of clothing. In a clinical setting, where rain or snow might intrude into the model during wrapping, the use of a vapor barrier may be beneficial even if wet clothing is removed.

In some cases, and under certain conditions, we believe the negative impact of increased exposure to severe weather conditions may not outweigh the observed advantages of the significantly increased rate of mean skin rewarming seen in the dry group compared to the wet group. However, an absolute prerequisite for this claim is a practiced, standardized method of clothing removal and wrapping to minimize exposure time [13]. In addition, there are possible scenarios in which this claim may be invalid; for example, if a shelter (ambulance, cabin, or other) is immediately available, it would make sense to move the patient to a shelter before removing clothes in order to minimize the patient’s exposure to a hostile environment.

In this study, the participants in the dry group reported that they felt slightly warmer and had a drier skin feeling after wrapping whereas those in the wet group felt that the chest and neck area was slightly wet during the entire experiment. There was a trend wherein the dry group felt warmer and more comfortable than the wet group. However, neither of these differences were significant, and the trends must be interpreted with caution.

The sensation of thermal comfort is linked to improved psychological and physiological status, and warmth contributes to feelings of comfort and safety [24]. The subjective feeling of being cold is an unpleasant sensation, and thermal discomfort in patients contributes to increased pain, fear, anxiety, an overall sense of dissatisfaction, and, eventually, fear of dying [25]. A qualitative study focusing on the experience of cold showed that shivering is particularly uncomfortable [8]. In a field study on the experiences of injured and ill patients with cold exposure, the authors found that cooling of the back and chest was the main cause of the overall sensation of discomfort [26].

This study did not detect any significant intergroup difference in terms of thermal comfort. Nonetheless, we speculate that the feeling of being dry and experiencing a faster T_skin_ increase will contribute to a favorable subjective experience, especially if the patients are colder than those in our study or if they are subject to fear or pain. Under harsh weather conditions, cold exposure during the removal of wet clothing may have affected patients more negatively than it did in the present study. To minimize exposure time, we recommend rehearsing the practice of rapid removal of clothing and wrapping patients in a model with a vapor barrier, insulation, and a wind- and waterproof outer layer.

In these patients, exposure during the prehospital phase is usually necessary to adequately assess for injury or illness. Although, in some cases, it might be appropriate to perform a primary survey after an initial evacuation of the patient, this will require the wrap to be “reopened” momentarily at a later stage for clinical examination and monitoring. If the patient is initially wrapped without removing wet clothing, a larger amount of water is phase-shifted to vapor, which immediately escapes when the model is reopened, thereby leading to a large loss of latent heat in the form of vapor from the model. The evaporative heat loss from opening the model later is most likely to be larger than the heat loss from a patient who has wet clothing removed before being placed in the model. Naturally, the amount of heat loss depends on how much water is retained in the wrapping model; for example, if the patient’s clothing is soaked wet or slightly wet. This further strengthens the claim that wet clothing should be removed as soon as possible, particularly if a patient’s clothes are extremely wet.

### Strengths and limitations

The present study enrolled healthy volunteers with normal thermoregulatory mechanisms, and the results may not be valid in all real-life scenarios. Age, potential trauma, and the physical condition of the patient may affect the rate of heat loss and thermoregulatory ability. Field conditions and the quality of care, such as weather conditions and duration of exposure during clothing removal, may also affect the rate of heat loss. Injured or ill patients may have had a worse experience of thermal discomfort with decreased temperature than the young, healthy, and motivated participants.

It is important to be aware of some limitations in transferability from our research setting to a real-life rescue scenario. In clinical practice, complete removal of clothing and insulation of the patient in less than 2.5 min may be difficult to achieve, especially without extensive training and preparation. Another important limitation of our study which may reduce clinical impact is the fact that that our research participants were dressed in cotton clothing, which may not be the most encountered material in victims of accidental hypothermia. Using cotton enabled us to achieve consistent levels of water saturation in the clothing, so we chose this to increase internal validity, possibly sacrificing some external validity.

Despite standardization, field studies may be biased by changes in temperature and wind. By conducting the study in a snow cave, we eliminated the impact of wind and limited the temperature fluctuations; however, the environment could not be fully controlled outside the laboratory setting. Temperature was not measured for the ‘warm bathroom floor’ or ‘room temperature’.

Blinding of the participants and researchers was not possible, which may have influenced the subjective scoring. However, we do not believe that this factor caused any systematic bias because the participants were not informed of the actual temperature recordings before or during the tests or of the assumed effects of the different treatment methods. Although the number of participants in this study was limited, the crossover design enabled a comparative evaluation of the interventions. However, systematic bias could not be completely excluded. In addition, the main analyses included a large number of tests (one per time point), and these tests are highly correlated with each other. Nevertheless, we believe that the results are transferrable to clinical settings because of the controlled and field-like experimental conditions.

The findings mentioned above may be relevant to how professional and volunteer search and rescue services are equipped and how they treat cold-stressed or hypothermic patients in the field, when combined with demanding environmental conditions. Future research should focus on real-life scenarios and on collecting data from patients with hypothermia.

## Conclusion

In a wind-sheltered cold environment, the benefit of removing wet clothes seems to outweigh the disadvantages of a brief exposure to a cold environment. Removing wet clothes may be preferable to retaining them beneath a vapor barrier in certain cases and under specific conditions.

### Electronic supplementary material

Below is the link to the electronic supplementary material.

**Supplementary Material 1**: Subjective questionnaire form

**Supplementary Material 2**: Subjective questionnaire answers and explanation

**Supplementary Material 3**: Temperature and humidity in the snow cave

## Data Availability

The datasets used and/or analyzed during the current study are available from the corresponding author on reasonable request.

## Abbreviations

95% CI: 95% confidence interval
°C: degree Celsius
T_core_: body core temperature
T_skin_: skin temperature
ANCOVA: analysis of covariance
