# Supplementary material for: Effect of wet clothing removal on skin temperature in subjects exposed to cold and wrapped in a vapor barrier: a human, randomized, crossover field study
Source: BMC Emerg Med. 2024 Jan 25;24:18. doi: 10.1186/s12873-024-00937-8 (PMC10809790; doi:10.1186/s12873-024-00937-8)
Supplement: Supplementary file 2 — Supplementary Material 2: Subjective questionnaire answers and explanation [file 12873_2024_937_MOESM2_ESM.pdf]

Subjective evaluation questionnaire

1. **How is the thermal sensation of your**
   a. Body
   b. Feet
   c. Hands
   d. Head
   e. Neck

   -5 Extremely cold
   -4 Very cold
   -3 Cold
   -2 Cool
   -1 Slightly cool
   0 Neutral
   1 Slightly warm
   2 Warm
   3 Hot
   4 Very hot
   5 Extremely hot

2. **Shivering/sweating. You are**

   -5 shivering extremely from cold
   -4 shivering a lot
   -3 shivering moderately
   -2 shivering slightly
   -1 barely shivering
   0 Neither shivering nor sweating
   1 barely sweating
   2 sweating slightly
   3 sweating moderately
   4 sweating a lot
   5 sweating extremely from heat

3. **How does your skin feel?**

   1 Drier than normal
   2 Normally dry
   3 Chest and back slightly wet
   4 Chest and back wet
   5 Whole body wet
   6 Whole body wet and clothing sticking to the skin

4. **How would you prefer your surrounding temperature?**

   -2 A lot cooler
   -1 Slightly cooler
   0 Neutral
   1 Slightly warmer
   2 A lot warmer

5. **How do you feel with regards to thermal comfort?**

   1 Comfortable
   2 Slightly uncomfortable
   3 Uncomfortable
   4 Very uncomfortable
