# Supplementary figures and images for: Effect of wet clothing removal on skin temperature in subjects exposed to cold and wrapped in a vapor barrier: a human, randomized, crossover field study
Source: BMC Emerg Med. 2024 Jan 25;24:18. doi: 10.1186/s12873-024-00937-8 (PMC10809790; doi:10.1186/s12873-024-00937-8)

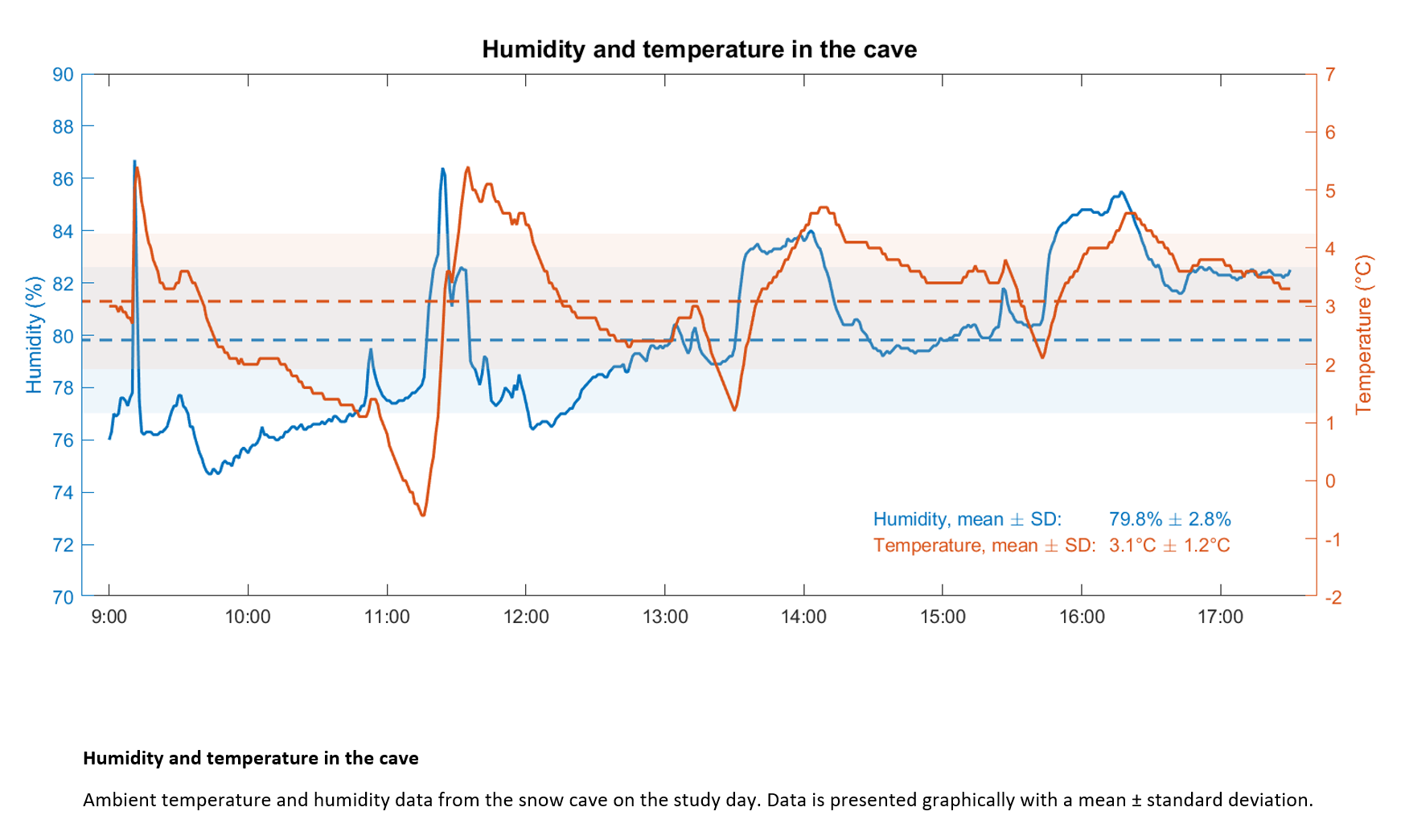

Supplement: Supplementary file 3 — Supplementary Material 3: Temperature and humidity in the snow cave [file 12873_2024_937_MOESM3_ESM.png]
